# Two Outbreaks of Occupationally Acquired Histoplasmosis: More than Workers at Risk

**DOI:** 10.1289/ehp.7484

**Published:** 2005-02-04

**Authors:** Gregory D. Huhn, Connie Austin, Mark Carr, Diana Heyer, Pam Boudreau, Glynnis Gilbert, Terry Eimen, Mark D. Lindsley, Salvatore Cali, Craig S. Conover, Mark S. Dworkin

**Affiliations:** ^1^Centers for Disease Control and Prevention, Atlanta, Georgia, USA;; ^2^Division of Infectious Diseases, Illinois Department of Public Health, Chicago, Illinois, USA;; ^3^Division of Infectious Diseases, and; ^4^Division of Environmental Health, Illinois Department of Public Health, Springfield, Illinois, USA;; ^5^Macon County Health Department, Decatur, Illinois, USA;; ^6^Kankakee County Health Department, Division of Chronic and Communicable Disease, Kankakee, Illinois, USA;; ^7^Ford-Iroquois Public Health Department, Watseka, Illinois, USA;; ^8^Mycotic Diseases Branch, Centers for Disease Control and Prevention, Atlanta, Georgia, USA;; ^9^University of Illinois Chicago, School of Public Health, Chicago, Illinois, USA

**Keywords:** antigen, bat guano, bridge, dust, histoplasmosis, landfill, occupationally acquired, spores, workers

## Abstract

Objective: The objective of this study was to determine the etiology and risk factors for acute histoplasmosis in two outbreaks in Illinois among laborers at a landfill in 2001 and at a bridge reconstruction site in 2003.

Design: We performed environmental investigations during both outbreaks and also performed an analytic cohort study among bridge workers.

Participants: Workers at the landfill during May 2001 and those at the bridge site during August 2003 participated in the study. At the landfill, workers moved topsoil from an area that previously housed a barn; at the bridge, workers observed bat guano on bridge beams.

Evaluations/Measurements: We defined a case by positive immunodiffusion serology, a ≥ 4-fold titer rise in complement fixation between acute and convalescent sera, or positive urinary *Histoplasma capsulatum* (HC) antigen. Relative risks (RR) for disease among bridge workers were calculated using bivariate analysis.

Results: Eight of 11 landfill workers (73%) and 6 of 12 bridge workers (50%) were laboratory-confirmed histoplasmosis cases. Three bridge workers had positive urinary HC antigen. At the bridge, seeing or having contact with bats [RR = 7.0; 95% confidence interval (CI), 1.1–43.0], jack-hammering (RR = 4.0; 95% CI, 1.2–13.3), and waste disposal (RR = 4.0; 95% CI, 1.2–13.3) were the most significant job-related risk factors for acquiring histoplasmosis.

Conclusions: Workers performing activities that aerosolized topsoil and dust were at increased risk for acquiring histoplasmosis.

Relevance to Professional and Clinical Practice: Employees should wear personal protective equipment and use dust-suppression techniques when working in areas potentially contaminated with bird or bat droppings. Urinary HC antigen testing was important in rapidly identifying disease in the 2003 outbreak.

Acute pulmonary histoplasmosis is a disease caused by *Histoplasma capsulatum* (HC), a dimorphic fungus commonly found in the United States in soil along the Mississippi and Ohio river valleys (Deepe 2000). Infection results primarily from inhalation of aerosolized spores from areas of high organic content in the soil. Bird or bat guano and rotting wood are classic reservoirs for *H. capsulatum* that can enrich soil matter to promote spore growth (Ajello 1964; Davies and Colbert 1990). Unlike bird droppings, bat guano may produce transmissible foci of fungi in the absence of soil conditions when physically disrupted (Ashford et al. 1999).

In Illinois, *H. capsulatum* endemnicity is high throughout most of the state, although in the northeast region near metropolitan Chicago, areas exist with relatively low endemic rates (Edwards et al. 1969). During 1991–2000, a median of 41 histoplasmosis cases was reported annually in Illinois; at least 14% of these cases were in immunocompromised persons. Histoplasmosis can reactivate in immunocompromised persons; therefore, it is uncertain how many case reports represented new infections versus reactivation of previous infections. In immunocompetent hosts, histoplasmosis is usually characterized by influenza-like symptoms with a duration of several weeks. Because illness is mild or self-limited in > 90% of cases, most sporadic infections elude diagnosis (Deepe 2000). The last epidemic of histoplasmosis in Illinois was recorded in 1983.

On 8 May 2001, the Macon County Health Department in central Illinois was notified of a hospitalized person with respiratory symptoms who was employed at a landfill in Macon County. By 10 May, five other workers from the landfill reported illness with similar symptoms of fever, chest pain, and shortness of breath. All six employees were heavy-equipment operators who moved topsoil at a trash deposition cell and cleared trees at a new landfill cell under construction. Serum specimens from the six workers tested positive for antibodies to *H. capsulatum* by immunodiffusion and complement fixation (CF) assays.

On 26 August 2003, the Ford-Iroquois Public Health Department (FIPHD) in east Illinois was notified of a cluster of five ill workers with fevers and respiratory symptoms at a bridge reconstruction site in Iroquois County. Onsets of illness for the five workers were 16–22 August. During 3–5 August, large concrete beams had been removed from one end of the bridge. Workers observed bat guano on the interior aspects of the extracted beam sections. On 29 August, the Illinois Department of Public Health (IDPH) was informed that urine specimens collected 24 hr earlier from all ill workers had positive tests in three of the five workers for *H. capsulatum* antigen by enzyme immunoassay (EIA).

In this article we summarize findings from investigations at the landfill and the bridge reconstruction sites. Each of these outbreaks highlights the need for histoplasmosis education for workers in high-risk occupations for *H. capsulatum* exposure. For the bridge investigation, we describe the value of rapid diagnostic tests to detect an acute histoplasmosis outbreak in a workplace and the use of test results to spur awareness of histoplasmosis among residents potentially at risk for infection in the surrounding community.

## Materials and Methods

### Case definition.

We defined a clinical case of acute histoplasmosis as an influenza-like illness (i.e., self-reported fever or chills plus one of the following symptoms: headache, chest pain, or shortness of breath) in workers employed at the landfill in Macon County with onset of symptoms during May 2001 or at the Iroquois County bridge reconstruction site with onset of symptoms during August 2003. A laboratory-confirmed case was defined as any worker, symptomatic or asymptomatic, who had laboratory evidence of recent *H. capsulatum* infection (CF antibodies to the mycelial and/or yeast-phase antigen at a titer of ≥ 1:32, H and/or M band by immunodiffusion, seroconversion of negative to positive H and/or M immunodiffusion band, a ≥ 4-fold rise in CF titer between acute and convalescent sera, or urinary *H. capsulatum* antigen > 1.0 EIA units) during the respective study periods.

### 2001 Landfill investigation.

#### Case finding.

Using a standardized questionnaire, IDPH and Macon County Health Department staff interviewed landfill workers employed by two companies (A and B) who were present at the site during the suspected exposure period. The questionnaire included questions regarding demographic information, symptoms of illness, job duties at the landfill site, underlying illness, state of residence, and treatment. Acute and convalescent sera were collected from all ill employees and tested for antibodies to *H. capsulatum* by immunodiffusion and CF at the Centers for Disease Control and Prevention (CDC, Atlanta, GA).

#### Environmental investigation.

We visually inspected the landfill site for areas of bird or bat roosting. We interviewed managers and owners for companies A and B to verify employee work shifts and activities. The land-fill was mapped into discrete areas and categorized according to the presence of bird or bat roosting sites, trash deposition, topsoil moving, dust suppression, and tree removal.

### 2003 Bridge investigation.

#### Case finding.

Using a standardized questionnaire, IDPH, Kankakee County Health Department (adjacent county north of Iroquois County), and FIPHD staff interviewed bridge workers employed during August 2003. The questionnaire included questions regarding demographic information (including number of years of residency in a state within the Mississippi or Ohio river valley), symptoms and duration of illness, job duties, bat or bat guano exposure, recreational activities with known risks of histoplasmosis exposure, underlying illness, history of previous histoplasmosis, treatment, and missed days of work. On 3 September 2003, the IDPH distributed a notice to infectious diseases physicians and infection control practitioners in the state recommending that the diagnosis of histoplasmosis be considered for patients with acute influenza-like illness who lived near or recently traveled to the area of the bridge site. FIPHD staff visited residents in homes in close proximity to the bridge site to assess whether symptoms compatible with histoplasmosis were present. FIPHD staff provided educational material about recognition and prevention of histoplasmosis to every bridge worker and community resident they visited.

All workers present at the bridge site at any time during 1–30 August comprised the full cohort in which we conducted a study to evaluate risk factors for histoplasmosis, including age, residency within the Mississippi or Ohio river valleys, preexisting illness, and specific job duties in persons with acute HC seroconversion. We screened workers with fever and respiratory symptoms for histoplasmosis infection by physical exam, urinary testing for *H. capsulatum* antigen, and acute and convalescent sera tests for antibodies to *H. capsulatum* by immunodiffusion and CF assays. We asked non-ill workers to submit acute sera for immunodiffusion and CF testing for antibodies to *H. capsulatum*.

#### Environmental investigation.

We visually inspected the bridge site for areas of bird and bat roosting and bat guano and observed workers for use of personal protective equipment, including respirators. We obtained hourly wind direction reports for 3–5 August from the Illinois Climate Network from the nearest meteorologic field station, approximately 25 miles from the bridge site. We also reviewed blueprints for the bridge reconstruction project to uncover potential occult bird or bat roosting areas. Approximately 1 g of bat guano was collected from a dismantled bridge beam and cultured for 7 days on malt extract and V-8 agar plates for histoplasmosis.

### Laboratory assays.

Blood samples were collected in serum separator tubes (Becton-Dickenson, Franklin Lakes, NJ), centrifuged for 15 min at 10,000 rpm, and shipped on ice to the CDC for testing. We obtained convalescent sera 19–21 days after the acute sera; these samples were tested in parallel with the acute sera. We tested serum specimens for antibodies to *H. capsulatum* by immunodiffusion and CF against mycelial and yeast-form antigens (Reiss et al. 2002). Urine samples were collected in sterile specimen containers, and we tested 0.1 mL of undiluted urine for *H. capsulatum* var *capsulatum* antigen detection using a polyclonal rabbit anti-histoplasma IgG antibody EIA (Durkin et al. 1997).

### Statistical analysis.

For the 2003 cohort study, we used Epi Info 6.04D (CDC, Stone Mountain, GA) to perform univariate and stratified analysis using 95% confidence intervals (CIs) and Fisher exact test when applicable to determine differences in potential risk factors between cases and noncases.

## Results

### 2001 Landfill investigation.

Among the 11 employees of company A, 6 met the clinical and laboratory-confirmed case definitions for histoplasmosis. Two additional employees had mild symptoms and did not meet the clinical case definition; however, both of these cases were laboratory confirmed. From company A, the attack rate for acute histoplasmosis cases was 73% (8 of 11). Nine of the 11 company A employees were from states outside the Mississippi and Ohio river valleys and started work in Illinois in mid-April 2001. Of the 7 employees of company B, none reported illness, and no specimens were tested for histoplasmosis. All employees of company B were local Macon County residents.

The dates of onset of illness for cases were 1–8 May 2001. The mean age of case patients was 31 years (range, 19–42 years), and all were men. Cough, fever or chills, and headache were the most commonly reported symptoms (Table 1). Two of the 11 case patients were hospitalized and treated with itraconazole. Four of the 5 case patients who had chest radiographs had evidence of lung nodules.

### 2001 Environmental study.

The 64-acre landfill is located in a rural area in central Illinois and started operations in the 1970s. The site is used for municipal and industrial waste. No substantial quantities of bird or bat droppings or other substantial quantities of animal waste are trucked into the site. Although birds were present at the site, no active bird roosts were identified. The manager of the landfill reported no known previous clusters of histoplasmosis among workers at the site.

All but one employee of company A were heavy-equipment operators. Work duties included moving topsoil in the active trash deposition site and in a new cell under construction, where a few large trees were knocked down and topsoil was scraped from a zone where an old barn had been located. Trucks carrying waste routinely dumped their contents at the center of the trash deposition site every few minutes, where it was compacted and covered by dirt brought in at night from the new cell by employees of company A. During 24–26 April, company A employees removed the trees from the new cell. Company B employees did not participate in the tree-removal activity, although they participated in moving and compacting soil in the active deposition site during this period.

### 2003 Bridge investigation.

Of the 12 workers at the bridge site, 5 reported illness compatible with the clinical case definition for histoplasmosis. All 5 ill workers had serologic evidence of acute histoplasmosis infection, and 3 of the 5 had positive detection for histoplasma antigen in urine collected on 28 August. One additional asymptomatic worker met the laboratory-confirmed case definition with a positive serology test for acute histoplasmosis infection. The attack rate for histoplasmosis cases at the bridge site was 50% (6 of 12).

The dates of onset of illness for cases were 14–22 August. The mean age of case patients was 47 years (range, 37–59 years), and all but one were men. Cough, fever or chills, sweats, fatigue, and headache were the most frequently reported symptoms and persisted ≥ 10 days in the five symptomatic workers (Table 1). One of the six case patients was hospitalized for 6 days, and three of five symptomatic case patients were treated with itraconazole. Four of the five symptomatic case patients had chest radiographs, and all four documented lung nodules or opacifications.

Among the five symptomatic workers, the mean time off from work was 21 days (range, 3–42 days). Two months after the initial outbreak, all five symptomatic workers had resolution of their symptoms, except for fatigue.

**Figure 1:**
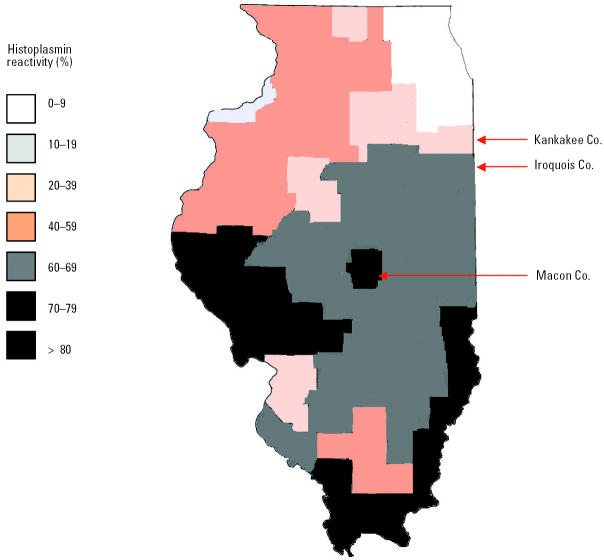
Histoplasmin reactivity by county in Illinois among naval recruits (*n* = 14,095). Figure modified from Edwards et al. (1969) with permission from the *American Review of Respiratory Disease*.

In the cohort study, all workers were residents of Illinois. Five of the six case patients were from Kankakee County, and all six of the non-case patients were from either Iroquois County or adjacent counties to the west of Iroquois County (Figure 1). The most significant risk factors for acquiring acute histoplasmosis were seeing or having contact with bats and/or bat guano at the bridge site [relative risk (RR) = 7.0; 95% CI, 1.1–43.0; *p* = 0.02) and residency in Kankakee County (RR = 7.0; 95% CI, 1.1–43.0; *p* = 0.02; Table 2). Job activities at the bridge site that approached a significant association with acquiring acute histoplasmosis included jack-or air-hammering (RR = 4.0; 95% CI, 1.2–13.3; *p* = 0.06) and waste disposal (RR = 4.0; 95% CI, 1.2–13.3; *p* = 0.06). Stratified analysis on risk factors was not possible because of zero cell calculations.

One resident of a trailer park near the bridge site was diagnosed with acute pulmonary histoplasmosis by a local physician, who had been alerted to the bridge worker outbreak by the statewide IDPH notification that occurred within 2 weeks of the start of the investigation. A previously healthy 35-year-old woman, who was a native of Iroquois County and lived approximately 30 yd from the southwest end of the bridge, reported onset of fever (measured temperature, 105°F), cough, headache, and myalgias on 6 September 2003. Urine collected on 9 September was weakly positive (1.31 EIA units) for histoplasma antigen. During August and early September, the woman had no long-distance travel outside her home and denied activities with known risks for *H. capsulatum* exposure (i.e., gardening, farming, or spelunking), but frequently fished from the embankment of the river below the bridge. No other residents in the 65 homes of the trailer park and surrounding community reported recent influenza-like symptoms after the door-to-door education campaign by FIPHD staff on 13 October.

### 2003 Environmental study.

The 378-ft, two-lane concrete bridge was built in 1979, and reconstruction started in January 2003 because parts of the bridge had prematurely lost structural integrity. All concrete beams and expansion joints were scheduled for replacement by steel support structures. By early June, all 12 employees were working on daily Monday–Friday shifts. On 4 July flood waters 18 ft over the embankment below the bridge forced labor crews to work only on the top section of the bridge. On 3–5 August, the flood waters receded, which allowed work crews to dismantle bridge beams on one lane of the bridge. A five-person crew composed of four laborers and one carpenter dislodged large sections of concrete beams on the north end of the bridge using jack- or air-hammers for extraction by crane operators. The operation produced considerable dust in the work zone. Water collected from the river in 5-gal buckets was used for dust suppression. The crew observed bats flying from gaps in the extracted beam sections. Surfaces of adjacent longitudinal bridge beams were stained with a dark film, and workers noted a heavy stench of ammonia. The five workers of the crew at the north end of the bridge were the only persons who became ill. On 3 August, the wind direction during working hours was primarily from the east or southeast. During working hours on 4–5 August, the wind direction was primarily from the north or northeast.

**Figure 2:**
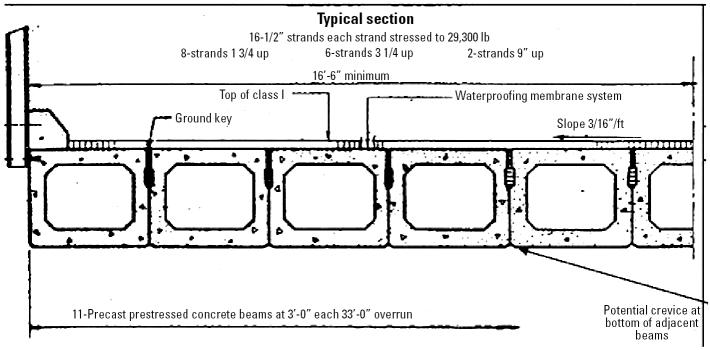
Blueprint cross-section longitudinal diagram of a precast, prestressed concrete bridge beam section associated with the development of histoplasmosis among workers at a bridge in Iroquois County, Illinois, in 2003.

Engineering blueprints of the bridge structure illustrated grout-filled transverse tie assemblies securing 3-ft concrete beam sections in a longitudinal grid pattern. The bridge beams were constructed with precast, prestressed concrete beams. Crevices between the beam sections of up to 1 ft were possible on the nongrouted bottom surface of the grid pattern if the beams were not manufactured longitudinally straight, if they became warped, or if small portions of concrete cracked and separated from a beam (Figure 2). A sample of bat guano collected and cultured from a dismantled bridge beam from the north end of the bridge was negative for *H. capsulatum*.

## Discussion

These two occupationally acquired outbreaks of acute pulmonary histoplasmosis illustrate point sources of intense *H. capsulatum* exposure in susceptible workers. At the landfill, large trees were uprooted and topsoil was moved from a previously unused site approximately 1–2 weeks before the ill workers reported symptoms. The topsoil, roughly 6–12 in. in depth, probably had *H. capsulatum* spore accumulation from the fallen trees and possibly from old bird roosts in the abandoned barn (Davies and Colbert 1990; Ward et al. 1979). Although the heavy-equipment operators who did not become ill worked exclusively in the area of trash deposition and were also exposed to this topsoil, they were probably not affected for several reasons: *a*) they moved additional dirt that was deeper beneath the topsoil than where *H. capsulatum* spores are normally found; *b*) they were not in the vicinity of the uprooted trees; *c*) and they were local residents of Macon County who might have had previous exposure to *H. capsulatum*, which provides partial immunity to reinfection (Goodwin and Dez Prez 1978).

For workers at the bridge site, case patients became ill within 2–3 weeks after dismantling beam sections on 3–5 August 2003, and bats had been observed by workers during the dismantling. Bats were potentially able to penetrate gaps from the bottom surface of the bridge joints and establish roosts; only the adjacent aspects of the bridge beams had evidence of bat guano. The primary responsibilities of the ill laborers on the five-person work crew were jack- or air-hammering and waste disposal. Both of these jobs are dust-inducing activities known to aerosolize bat guano [National Institute for Occupational Safety and Health (NIOSH) 1997] and were the greatest job-duty risk factors identified in this investigation.

Symptomatic disease in immunocompetent hosts usually correlates with a high inoculum of *H. capsulatum* spores (Wheat 2001). The one asymptomatic worker, a carpenter, may have inhaled a lower inoculum of spores because his job did not involve direct aerosolization of dust. The ill workers might have also lacked partial immunity to histoplasmosis because most were long-time residents of counties in Illinois where the estimated seropositivity of histoplasmosis is lower than in the central portions of the state (Edwards et al. 1969). Areas bordering endemic regions for *H. capsulatum* are common points of outbreaks of symptomatic histoplasmosis in susceptible persons with *H. capsulatum* exposure (Gustafson et al. 1981; Schoenberger et al. 1988; Ward et al. 1979). Historically, histoplasmin reactivity in a representative Kankakee County population has been 20–39% versus 60–69% in populations in central Illinois (Edwards et al. 1969).

For > 50 years, occupational risks of histoplasmosis in bridge workers have been well documented (Englert and Phillips 1953; Jones et al. 1999; Sorley et al. 1979). Although water was poured on the bridge beams for dust suppression during jack- or air-hammering, the workers at the bridge site rarely wore respirators. Well-fitted N95 respirators, ubiquitous in airborne infection control practices, can filter *H. capsulatum* spores (particles of 1–2 μ m) (NIOSH 1997). *H. capsulatum* can colonize in the gastrointestinal tracts of bats, obviating the requirement in some settings for a soil reservoir in the transmission of spores from bat guano (Hoff and Bigler 1981). This outbreak demonstrates that typical design features of concrete bridges (Volle 2002) can support occult areas of *H. capsulatum* exposure, further underscoring the importance of respirator use even in the absence of bat roosts or guano by routine visual inspection.

Acute pulmonary histoplasmosis may manifest symptoms over several weeks to months. The average duration of time off work for the ill bridge workers was 3 weeks. The economic impact of possible loss of production should also be considered in addition to worker safety in promoting education and training programs regarding risks for histoplasmosis in high-risk occupations.

In both of these outbreaks, we made general preventive recommendations to employers to reduce the risk of *H. capsulatum* exposure in the workplace. We encouraged risk assessment in consultation with an industrial hygienist or occupational safety agency before work in areas with current or historic bird or bat roosts. We also recommended deep burial of topsoil and waste suspected of *H. capsulatum* contamination. Worker training programs were encouraged to emphasize the use of appropriate personal protective equipment and water saturation for dust suppression during the mechanical removal of potentially contaminated material. Interruption of work activities during windy conditions was advised to minimize dust aerosolization (NIOSH 1997). Education regarding recognition of signs and symptoms of acute histoplasmosis, which might facilitate early diagnosis of cases, was also stressed. No further cases of histoplasmosis were reported among workers at these sites after the initial outbreaks.

There were limitations in these investigations that may have restricted our findings of asymptomatic or subclinical disease in workers. Asymptomatic landfill workers were not tested for acute histoplasmosis infection, and asymptomatic bridge workers with negative acute serologic results did not submit serum for convalescent testing to assess for *H. capsulatum* seroconversion. The small sample size of the cohort study of bridge workers also precluded analysis of independent risk factors for acquiring histoplasmosis. The environmental investigation at the bridge was also limited by the amount of bat guano that was available for retrieval from a discarded dismantled bridge beam. The quantity collected was insufficient to perform the gold standard test for isolation of *H. capsulatum* by mouse inoculation.

Serologic tests for acute pulmonary histoplasmosis are helpful to confirm the diagnosis, particularly in patients with mild disease by collection of both acute and convalescent sera; this is true because antibodies usually require at least 1 month to appear after the initial exposure (Wheat 2001). When immunodiffusion and CF serology tests are performed appropriately, their sensitivities are 75% and 95%, respectively, in patients with acute histoplasmosis. Immunodiffusion tests are simpler to perform and more specific than CF tests, but their lower sensitivity limits their usefulness as screening tests (Davies and Sarosi 1987). In the bridge investigation, we screened ill bridge workers for *H. capsulatum* antigen in urine within 2 days of first notification of this cluster. In immunocompetent hosts with acute pulmonary disease, sensitivity for antigen detection is > 80% (Wheat et al. 2002). These antigen test results were available within 24 hr of their collection and were positive in 60% of ill workers. The rapid turnaround time of these tests was important in establishing histoplasmosis as the cause of disease in this cluster. This information was critical in galvanizing rapid public health action for control of histoplasmosis in this community.

Workplace outbreaks of histoplasmosis can be sentinel markers for disease activity in the community. This was apparent in the 2003 outbreak when a resident in a nearby neighborhood was diagnosed with acute histoplasmosis after clinicians in the area were notified of the cluster of ill bridge workers. The resident’s house was downwind during most of the bridge beam dismantling, which might have produced aerosolized *H. capsulatum* spores. Windborne transmission of histoplasmosis has been implicated in certain areas and may contribute to prolonged outbreaks (D’Alessio et al. 1965; Leznoff et al. 1964; Sellers et al. 1965; Tosh et al. 1966; Wheat et al. 1981). Educational efforts should target not only workers in high-risk occupations, but also residents and health care providers in surrounding communities to promote awareness of the risks of histoplasmosis.

## Figures and Tables

**Table 1:** Symptoms of histoplasmosis [number (%)] in outbreaks among workers at a landfill in Illinois in 2001, and at a bridge in Illinois in 2003.

Symptom	2001 (*n* = 8)	2003 (*n* = 6)
Cough	7 (88)	5 (83)
Fever or chills	6 (75)	5 (83)
Sweats	2 (25)	5 (83)
Headache	6 (75)	5 (83)
Difficulty breathing	5 (63)	4 (67)
Fatigue	Unknown	5 (83)
Myalgias	Unknown	4 (67)
Anorexia	Unknown	4 (67)
Weight loss	Unknown	4 (67)
Chest pain	4 (50)	4 (67)
Diarrhea	2 (25)	2 (33)
Joint pain	3 (38)	3 (50)
Hemoptysis	Unknown	0 (0)
Rash	Unknown	0 (0)

**Table 2:** Risk factors associated with histoplasmosis among workers at a bridge in Illinois in 2003 (*n* = 12).

Characteristic	Total exposed	Cases [no. (%)]	RR (95% CI)	*p*-Value
Mean age (range)	12	47 (37–59)	NA	0.48
Male sex	11	5 (45)	0.5 (0.2–0.9)	1.0
History of smoking	7	4 (57)	1.4 (0.4–4.9)	1.0
History of histoplasmosis	0	0 (0)	Undefined	NA
Resident of state in Mississippi or Ohio river valley	12	6 (50)	Undefined	NA
Resident of Kankakee County, Illinois	5	5 (100)	7.0 (1.1–43.0)	0.02
Excavation	3	1 (33)	0.6 (0.1–3.3)	1.0
Plowing/digging	6	4 (67)	2.0 (0.6–7.1)	0.57
Sandblasting	2	2 (100)	2.5 (1.8–5.3)	0.45
Jack- or air-hammering	4	4 (100)	4.0 (1.2–13.3)	0.06
Carpentry	3	3 (100)	3.0 (1.2–7.6)	0.18
Finishing	2	1 (50)	1.0 (0.2–4.6)	1.0
Waste disposal	4	4 (100)	4.0 (1.2–13.3)	0.06
Worked on north side of bridge	11	6 (55)	Undefined	1.0
Inhaled dust	10	6 (60)	Undefined	0.45
Saw or had contact with bats	5	5 (100)	7.0 (1.1–43.0)	0.02
Contact with bat guano	4	4 (100)	4.0 (1.2–13.3)	0.06

NA, not applicable.
